# Central Role of Pyrophosphate in Acellular Cementum Formation

**DOI:** 10.1371/journal.pone.0038393

**Published:** 2012-06-04

**Authors:** Brian L. Foster, Kanako J. Nagatomo, Francisco H. Nociti, Hanson Fong, Daisy Dunn, Anne B. Tran, Wei Wang, Sonoko Narisawa, Jose Luis Millán, Martha J. Somerman

**Affiliations:** 1 Laboratory of Oral Connective Tissue Biology, National Institute of Arthritis and Musculoskeletal and Skin Diseases (NIAMS), National Institutes of Health (NIH), Bethesda, Maryland, United States of America; 2 Department of Periodontics, University of Washington School of Dentistry, Seattle, Washington, United States of America; 3 Division of Periodontics, School of Dentistry at Piracicaba, State University of Campinas, Piracicaba, São Paulo, Brazil; 4 Materials Science and Engineering, University of Washington, Seattle, Washington, United States of America; 5 Sanford Children's Health Research Center, Sanford-Burnham Medical Research Institute, La Jolla, California, United States of America; University of Southern California, United States of America

## Abstract

**Background:**

Inorganic pyrophosphate (PP_i_) is a physiologic inhibitor of hydroxyapatite mineral precipitation involved in regulating mineralized tissue development and pathologic calcification. Local levels of PP_i_ are controlled by antagonistic functions of factors that decrease PP_i_ and promote mineralization (tissue-nonspecific alkaline phosphatase, *Alpl*/TNAP), and those that increase local PP_i_ and restrict mineralization (progressive ankylosis protein, ANK; ectonucleotide pyrophosphatase phosphodiesterase-1, NPP1). The cementum enveloping the tooth root is essential for tooth function by providing attachment to the surrounding bone via the nonmineralized periodontal ligament. At present, the developmental regulation of cementum remains poorly understood, hampering efforts for regeneration. To elucidate the role of PP_i_ in cementum formation, we analyzed root development in knock-out (^−/−^) mice featuring PP_i_ dysregulation.

**Results:**

Excess PP_i_ in the *Alpl^−/−^* mouse inhibited cementum formation, causing root detachment consistent with premature tooth loss in the human condition hypophosphatasia, though cementoblast phenotype was unperturbed. Deficient PP_i_ in both *Ank* and *Enpp1* ^−/−^ mice significantly increased cementum apposition and overall thickness more than 12-fold vs. controls, while dentin and cellular cementum were unaltered. Though PP_i_ regulators are widely expressed, cementoblasts selectively expressed greater ANK and NPP1 along the root surface, and dramatically increased ANK or NPP1 in models of reduced PP_i_ output, in compensatory fashion. *In vitro* mechanistic studies confirmed that under low PP_i_ mineralizing conditions, cementoblasts increased *Ank* (5-fold) and *Enpp1* (20-fold), while increasing PP_i_ inhibited mineralization and associated increases in *Ank* and *Enpp1* mRNA.

**Conclusions:**

Results from these studies demonstrate a novel developmental regulation of acellular cementum, wherein cementoblasts tune cementogenesis by modulating local levels of PP_i_, directing and regulating mineral apposition. These findings underscore developmental differences in acellular versus cellular cementum, and suggest new approaches for cementum regeneration.

## Introduction

The mineralized tissues of the teeth and skeleton are subject to homeostasis of inorganic phosphate (P_i_) for normal development and maintenance [1]. The hydroxyapatite (HAP) deposited to mineralize these hard tissues is a compound of P_i_ and ionic calcium. Pyrophosphate (PP_i_), composed of two molecules of P_i_, functions as a pivotal regulator of physiological mineralization and pathologic calcification by acting as a potent inhibitor of HAP crystal precipitation [2]–[5]. Though the potential for PP_i_ to inhibit biological mineralization is clear from *in vitro* experiments, the *in vivo* role and regulation of PP_i_ has been more difficult to elucidate. Through study of the heritable conditions such as hypophosphatasia (HPP), spontaneous mutations, and directed gene ablations in mouse models, the key regulators of PP_i_ have been identified, and their roles in shaping mineralized tissues have been partially defined. As measurement of PP_i_ *in vivo* at mineralization fronts is not possible, the analysis of cellular proteins that manufacture, transport, or degrade PP_i_ has served to clarify the mechanisms for PP_i_ modulation, in conjunction with *in vitro* experiments.

**Figure 1:**
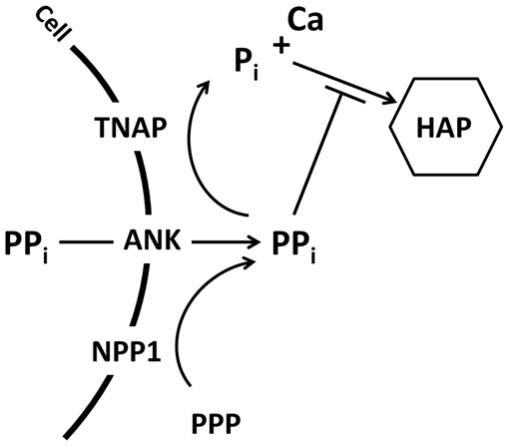
Pyrophosphate homeostasis in the extracellular space. Inorganic phosphate (P_i_) is a component of mineral hydroxyapatite (HAP), while pyrophosphate (PP_i_) is a potent inhibitor of HAP crystal precipitation and growth. The enzyme tissue nonspecific alkaline phosphatase (TNAP) hydrolyzes PP_i_ to release ionic P_i_, creating conditions conducive for mineralization. Local PP_i_ is increased by the functions of the progressive ankylosis protein (ANK) and ectonucleotide pyrophosphatase phosphodiesterase 1 (NPP1), which act to keep the mineralization process in check.

Local tissue concentrations of PP_i_ are controlled by a number of regulatory enzymes and transporters. Tissue nonspecific alkaline phosphatase (TNAP) is an ectoenzyme capable of hydrolyzing PP_i_ and providing P_i_ [6]. TNAP is expressed by mineralizing cells of bones and teeth, and is critical for proper skeletal mineralization [5], [7]. Hydrolysis by alkaline phosphatase activity (ALP) thus provides a mechanism for clearance of PP_i_, allowing mineralization to proceed. Loss of function mutations in the TNAP gene *Alpl* cause hypophosphatasia (HPP), a disease marked by poor bone mineralization, rickets, and osteomalacia, as well as tooth phenotypes [8], [9]. Ablation of the homologous mouse gene *Alpl* (formerly *Akp2*) produces a phenotype consistent with increased PP_i_ and mineralization disorders of infantile HPP [10], [11]. Conversely, two factors have been identified which increase local PP_i_ in tissues. The progressive ankylosis gene (*Ank*; *Ankh* in humans) encodes a multipass transmembrane protein that regulates transport of intracellular PP_i_ to the extracellular space [12]–[14]. Ectonucleotide pyrophosphatase phosphodiesterase 1 (NPP1; encoded by the *Enpp1* gene) also works to increase extracellular PP_i_ by hydrolysis of nucleotide triphosphates [15]. PP_i_ removal by ALP activity thus antagonizes provision of PP_i_ by ANK and NPP1, thereby creating a concerted regulation of P_i_ and PP_i_ levels (Figure 1), and ultimately, mineralization [16], [17].

The cementum covering the tooth root provides attachment for the tooth proper to surrounding alveolar bone, via the non-mineralized periodontal ligament (PDL) [18]–[20]. Cementum was first linked to PP_i_ metabolism by the condition HPP, where premature tooth exfoliation was discovered to result from developmental cementum aplasia or hypoplasia, and thus poor periodontal attachment [7], [21], [22]. Intriguingly, studies to date suggest the acellular cementum (acellular extrinsic fiber cementum, AEFC) of the cervical portion of the root is severely affected by PP_i_ dysregulation, while the apically located cellular cementum (cellular intrinsic fiber cementum, CIFC) is unaffected, or much less so [21], [23]. Proper cementum formation is critical for dentoalveolar function, though cementogenesis remains poorly understood in terms of associated cells and regulatory factors involved. This is especially true in regard to differences between the acellular and cellular varieties, and how cementum differs developmentally from other hard tissues, bone and dentin [18], [19].

To address how the process of cementogenesis is shaped by PP_i_ metabolism, a set of studies was designed that employed *in vivo* transgenic mouse models featuring disrupted PP_i_ regulation, as well as *in vitro* approaches using a cementoblast cell line for further mechanistic studies.

## Results

In order to develop a comprehensive understanding of how PP_i_ regulates tooth root development, we performed a detailed histological study of developing first mandibular molars and incisors of mice harboring homozygous knock-out (^−/−^) of *Alpl* (high PP_i_), *Ank*, or *Enpp1* (low PP_i_), compared to age-matched homozygous wild-type (^+/+^) controls. Days were selected to capture developmental time points of interest during molar root formation, i.e., during acellular cementogenesis (14 days postnatal, dpn), at completion of the root and following cellular cementogenesis (26 dpn), and after more than a month in occlusion (60 dpn). *Alpl* ^−/−^ mice were limited to a maximum age of 21 dpn because of shortened lifespan. Morphological observations on H&E stained sections were paired with *in situ* hybridization (ISH) and immunohistochemistry (IHC) for selected mineralized tissue-associated factors.

### Acellular cementogenesis requires diminution of pyrophosphate

**Figure 2:**
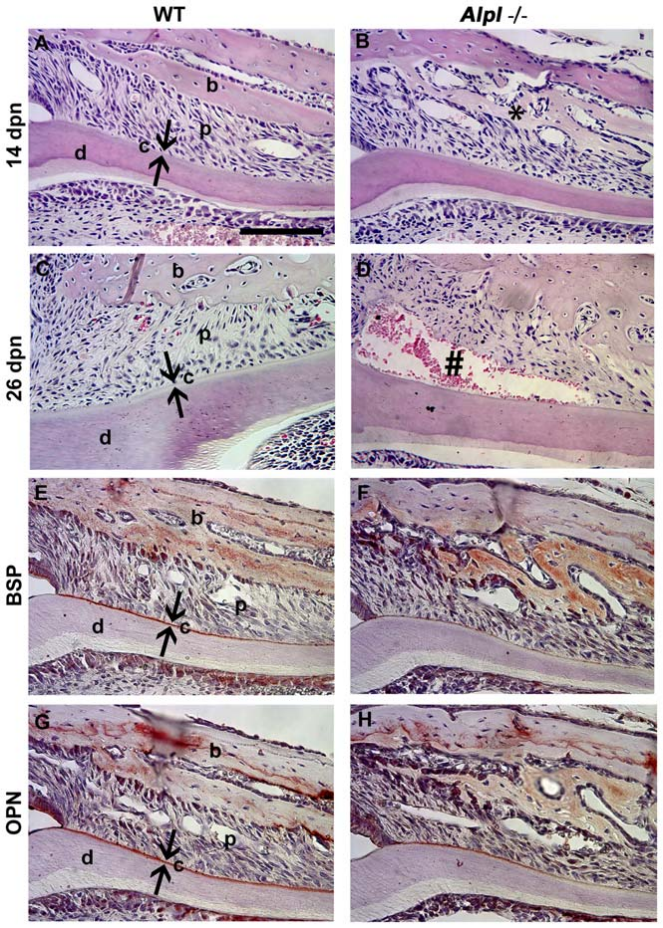
Acellular cementogenesis requires diminution of pyrophosphate. The *Alpl* ^+/+^ control first molar root at (A) 14 dpn and (C) 21 dpn, shows a normal periodontal architecture with a continuous layer of basophilic cementum (c) covering the root dentin (d) surface. In *Alpl* ^−/−^ molars, ablation of TNAP resulted in (B) hyperosteoidosis (*) and loss of the acellular cementum layer, and (D) a weak cementum-PDL interface, manifested by tearing (#). (E–H) Disrupted localization of cementum markers bone sialoprotein (BSP) and osteopontin (OPN) compared to control supported histological observations of cementum hypoplasia in 14 dpn *Alpl* ^−/−^ mouse molars. Abbreviations: d = dentin; c = acellular cementum; p = periodontal ligament; b = bone. Scale bar = 100 µm.

In the infantile form of HPP, the skeleton is properly mineralized at birth, but postnatal skeletogenesis is compromised [7]. *Alpl* ^−/−^ mice phenocopy aspects of infantile HPP, where loss of TNAP was previously reported to have little effect on bone until postnatal day 6 [10], [24]. At 14 dpn, the majority of alveolar and mandibular bone in *Alpl* ^−/−^ mice was well developed, though signs of hyperosteoidosis were noted in the bone adjacent to the molar root (Figure 2A and B). In *Alpl* ^+/+^ molars, acellular cementum (AEFC) covered the root dentin as a thin and uniform basophilic layer. *Alpl* ^−/−^ molars were marked by disruption of acellular cementum, visible as reduction of the basophilic layer (cementum aplasia or severe hypoplasia) and direct contact of PDL cells and tissues with dentin. By 21 dpn this cementum defect was sometimes associated with tearing at the PDL-AEFC interface, suggesting poor integration of Sharpey's fibers at the root surface (not seen at the PDL-bone interface) (Figure 2C and D) and consistent with HPP case reports observing premature tooth exfoliation. This is not likely to be a processing artifact, as infiltrating cells were present in the tear zone. These results agree with AEFC disruption described in this *Alpl* ^−/−^ model [25], as well as a different TNAP loss-of-function mouse [23].

**Figure 3:**
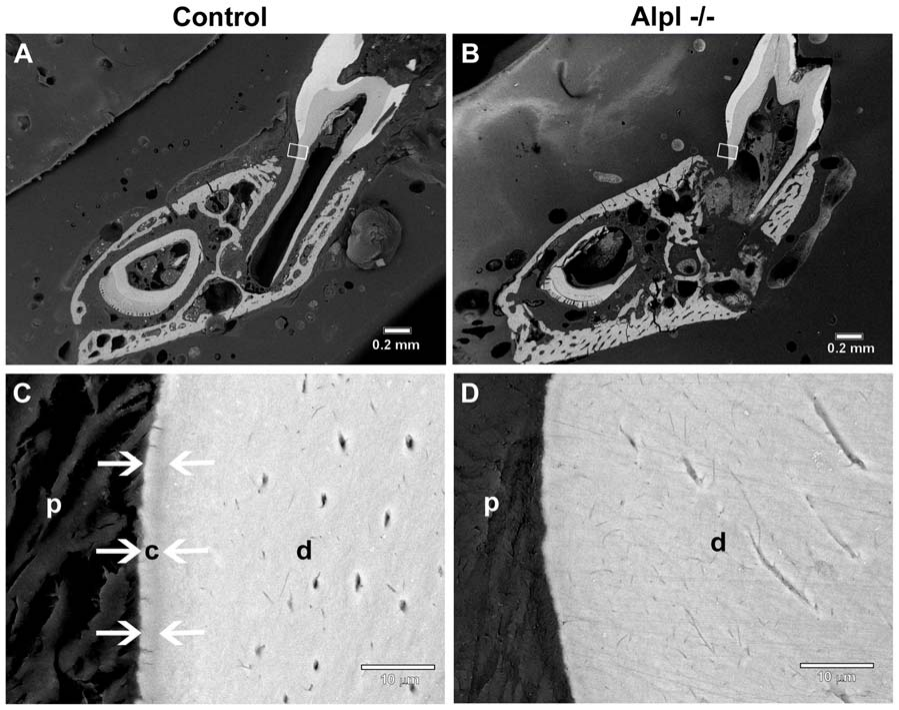
Lack of acellular cementum on *Alpl* ^−/−^ molar root surfaces. Backcattered SEM was employed to explore the cervical root surface (white boxes) in (A) *Alpl* ^+/+^ control and (B) *Alpl* ^−/−^ first molars. At higher magnification, the acellular cementum layer (white arrows) in the (C) control molar can be distinguished by contrast differences due to slightly lower mineralization than underlying dentin (d). (D) No acellular cementum layer was apparent in the cervical region of the *Alpl* ^−/−^ molar. Abbreviations: d = dentin; c = acellular cementum; p = periodontal ligament.

To further investigate the mechanism for the cementum defect in *Alpl* ^−/−^ mice, IHC was performed for two cementum markers, extracellular matrix (ECM) proteins bone sialoprotein (BSP) and osteopontin (OPN), which are present at high concentrations in acellular cementum of controls (Figure 2E and G). Both BSP and OPN immune localization were disrupted on the *Alpl* ^−/−^ root surface (Figure 2F and H), compared to the strong, even staining on *Alpl* ^+/+^ controls. Scanning electron microscopy (SEM) provided improved resolution to explore the root surface. While Alpl^+/+^ molars displayed a cementum layer on the root dentin surface, this layer was absent in the *Alpl* ^−/−^ molar (Figure 3). The disruption of cementum initiation and concomitant lack of BSP and OPN localization supports the hypothesis that high PP_i_ in *Alpl* ^−/−^ is acting to inhibit cementogenesis and HAP apposition on the root surface.

### Attenuation of pyrophosphate increases acellular cementum

**Figure 4:**
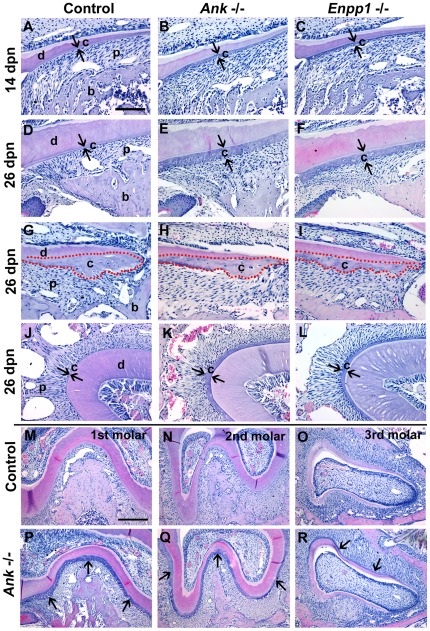
Attenuation of pyrophosphate increases acellular cementum. The cervical cementum (c) is a thin, acellular layer in *Ank*; *Enpp1* ^+/+^ control molars at (A) 14 dpn and (D) 26 dpn, while the (G) apical cementum is thicker and contains cementocytes. Knock-out of either *Ank* or *Enpp1* results in expanded cervical cementum compared to control, visible by 14 dpn (B and C), and progressively thicker by 26 dpn (E and F). In contrast, the apical cementum in *Ank* and *Enpp1* ^−/−^ molars (H and I) was not different from ^+/+^ control. (J–L) Acellular cementum of the incisor lingual root analog was similarly expanded in *Ank* and *Enpp1* ^−/−^ vs. control. (M–R) Hypercementosis resulting from loss of ANK was confirmed on all three mandibular molars. Abbreviations: d = dentin; c = acellular cementum; p = periodontal ligament; b = bone. Scale bar for A–L represents 200 µm, and for M–R represents 400 µm.

Both *Ank* and *Enpp1* ^−/−^ mice are deficient in extracellular PP_i_, though by different mechanisms. In molars of both null mice at 14 dpn, the developing cervical cementum was expanded (hypercementosis) compared to *Ank* and *Enpp1* ^+/+^ controls (Figure 4A–C). At the completion of root development at 26 dpn, both *Ank* and *Enpp1* ^−/−^ molars featured a nearly identical cementum phenotype where cervical cementum width was expanded several fold over controls (Figure 4D–F). This thick cervical cementum included numerous cell inclusions in the matrix, in a region that is typically acellular type cementum (AEFC). Intriguingly, for both homozygous knock-out models, apical cementum (CIFC) was not morphologically different from controls (Figure 4G–I), PDL space remained unmineralized, and dentin was not altered compared to *Ank* and *Enpp1* ^+/+^ mice.

The incisor in the mouse is divided into a (labial) crown analogue featuring enamel, and a (lingual) root analogue featuring strictly AEFC type cementum. Notably, histological changes in *Ank* and *Enpp1* ^−/−^ incisors paralleled those in molars, featuring expanded cementum (Figure 4J–L). Sagittal sections of the mandible allowed observation of all three molars. Loss of *Ank* affected all molars similarly, with thickened cementum evident on all root surfaces compared to controls (Figure 4M–R). The fact that acellular cementum on all murine teeth was similarly affected by reduced PP_i_ supports this as a central molecular regulator of cementogenesis which is not tooth- or stage-specific in its influence.

**Figure 5:**
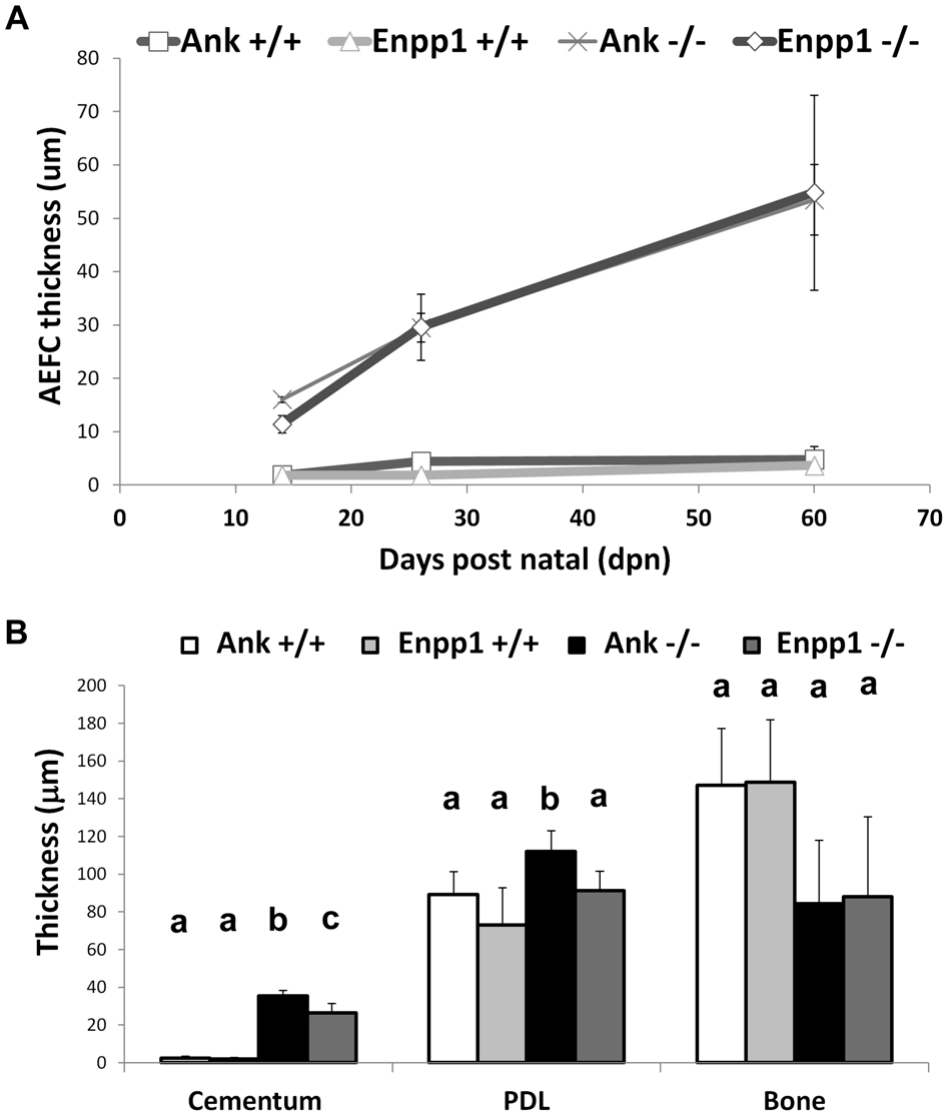
Increased cementum apposition in *Ank* and *Enpp1* ^−/−^ teeth. (A) During early root formation between 14 and 26 dpn, both *Ank* and *Enpp1* ^−/−^ molars featured at least 10-fold greater cementogenesis compared to controls. From 26 to 60 dpn, *Ank* and *Enpp1* ^−/−^ cementum continued to increase at a rate of 0.2–0.7 µm/day, while *Ank* and *Enpp1* ^+/+^ controls featured tightly controlled apposition at the pace of 0.01–0.05 µm/day. (B) Histomorphometry confirmed *Ank* or *Enpp1* ^−/−^ cervical cementum was significantly increased compared to controls, while PDL width was maintained and alveolar bone thickness tended towards reduction. Values with the same letter were not significantly different, while different letters indicate a statistically significant intergroup (genotype) difference (p<0.05) as tested by ANOVA followed by the Tukey test for direct pair-wise comparisons.

Both *Ank* and *Enpp1* ^−/−^ mice featured a hypercementosis phenotype, indicating both PP_i_ regulators function in controlling cementum formation. Comparative analysis between *Ank* and *Enpp1* ^−/−^ and their respective controls was accomplished by measuring the growth rate of cervical cementum over time. During early root formation between 14 and 26 dpn, *Ank* and *Enpp1* ^−/−^ molars featured at least 10-fold greater cementogenesis compared to controls (Figure 5A). *Ank* and *Enpp1* ^−/−^ cementum continued to increase at a rate of 0.2–0.7 µm/day from 26 to 60 dpn, while over the same period, controls featured tightly controlled apposition, growing at the much slower pace of 0.01–0.05 µm/day.

While cementum was dramatically affected by loss of ANK or NPP1, dramatic changes in other tissues were not observed. Histomorphometry at age 26 dpn was performed to measure cross-sectional widths to determine if PDL and alveolar bone were affected. Cementum was significantly increased in both null models, with *Ank* ^−/−^ at 14-fold and *Enpp1* ^−/−^ at more than 13-fold the width of age-matched controls (Figure 5B). A direct comparison of the two homozygous knock-out models revealed that *Ank* ^−/−^ featured slightly, but significantly, thicker cementum at the age sampled. Histomorphometry confirmed that PDL space was maintained in both null models, even significantly larger in *Ank* ^−/−^ vs. ^+/+^, despite exuberant cementogenesis. Alveolar bone on the lingual aspect tended towards reduced cross sectional dimension in both *Ank* and *Enpp* ^−/−^ models, though the effect was not statistically significant as measured here. Tartrate resistant acid phosphatase (TRAP) staining confirmed increased numbers of osteoclast-like cells (TRAP positive, multinucleated) on the bone surface adjacent to the tooth root in *Ank* ^−/−^ molars [26]. A modeling/remodeling of bone away from the root provides a mechanism for maintenance of the PDL in the face of expanding cementum.

**Figure 6:**
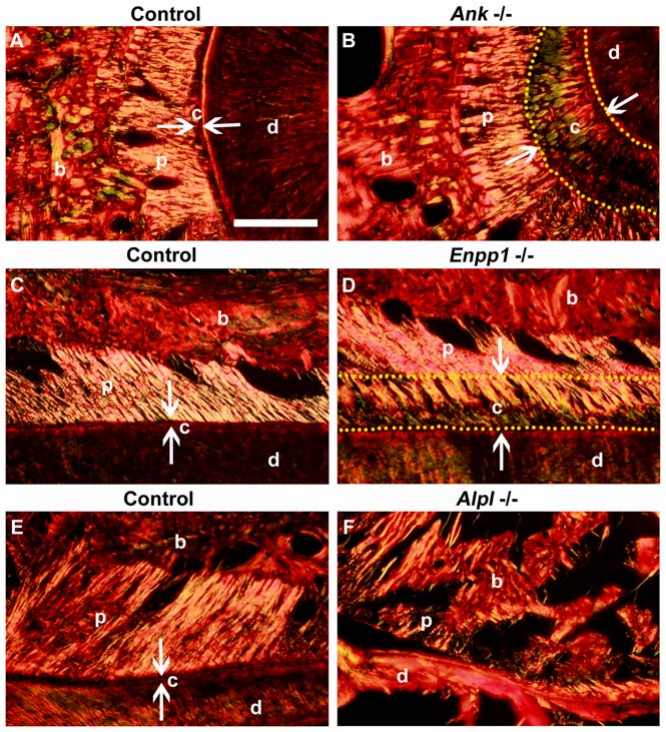
Progressive mineralization of extrinsic collagen fibers in *Ank* and *Enpp1* ^−/−^ cervical cementum. Picrosirius red staining with polarized light microscopy was used to visualize birefringent collagen fibers of periodontal tissues in mandibular first molar roots. Histological sections of 60 dpn (A) control *Ank; Enpp1* ^+/+^ cut in a horizontal plane and (C) coronal plane revealed high density of embedded extrinsic fibers in the acellular cementum, where the high degree of birefringence (intense coloration) makes visible the organization and orientation of the major PDL collagen fibers. Observation of (B) *Ank* ^−/−^ and (D) *Enpp1* ^−/−^ expanded cervical cementum (yellow dotted outline, flanked by white arrows) in the same orientations revealed a similar high density of embedded fibers, continuous from PDL through the cementum. (E) Control *Alpl* ^+/+^ molars at 21 dpn cut in a coronal plane show an organized and attached PDL, while conversely, (F) *Alpl* ^−/−^ molars exhibited tearing at the root-PDL interface (#), osteoid invasion of the PDL space, and poorly organized and sparsely embedded collagen fibers at the cervical root. Abbreviations: d = dentin; c = acellular cementum; p = periodontal ligament; b = bone. Scale bar = 100 µm.

One of the key functional characteristics of the cervical cementum is the extrinsic nature of the collagen fibers, which serve to anchor the tooth to surrounding alveolar bone. Picrosirius red staining in association with polarized light microscopy was used to visualize the birefringent collagen fibers of the periodontia [27]. The thick cementum of *Ank* and *Enpp1* ^−/−^ molars featured a high concentration of extrinsic collagen fibers, which were continuous with the fibers in the PDL proper (Figure 6B and D). As this thick cementum in the null molars features dense extrinsic collagen fibers, but also contains numerous cell inclusions, it could properly be labeled cellular extrinsic fiber cementum (CEFC), a form of cementum not typical for cervical molar roots, and furthermore, not previously described in the cementum family. Importantly, the observation of an ongoing, progressive apposition on the root surfaces of *Ank* and *Enpp1* ^−/−^ mice confirms this is thickening of the normally present extrinsic fiber cementum, and is not likely to be a different type of ectopic calcification on the root surface. As a comparison, *Alpl* ^−/−^ molars were examined, and confirmed tearing at the root-PDL interface, osteoid invasion of the PDL space, and poorly organized and sparsely embedded collagen fibers at the cervical root (Figure 6F).

**Figure 7:**
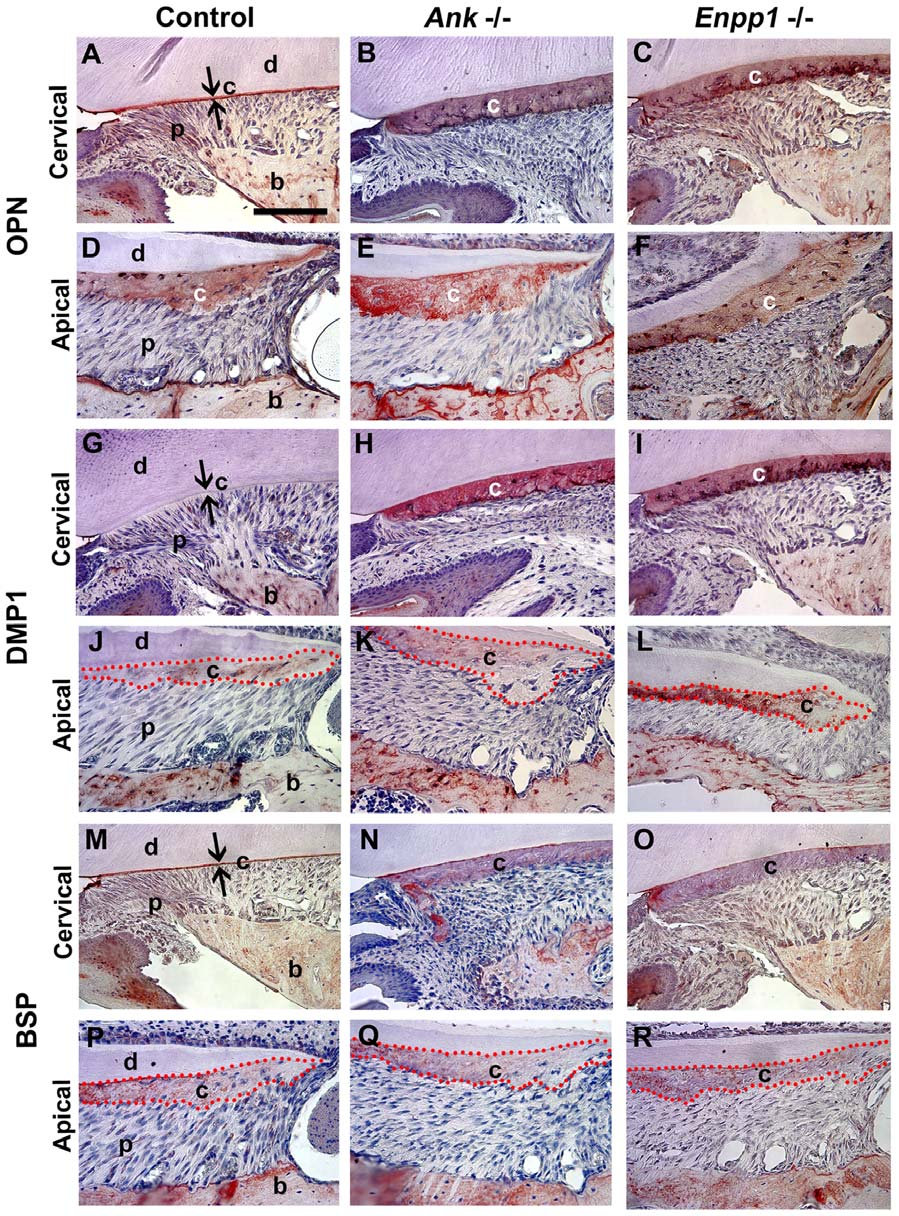
Reduced pyrophosphate alters acellular cementum matrix composition. IHC was performed on *Ank* and *Enpp1* ^+/+^ (Control) and ^−/−^ tissues at 26 dpn. OPN defines the acellular cementum layer in (A) wild-type cervical cementum, and is intensely localized to the thick AEFC in (B, C) both ^−/−^ models. DMP1 did not label acellular cementum in (G) ^+/+^ controls, but was increased dramatically in the thickened cervical cementum of (H, I) both ^−/−^ models. BSP was present in (M) control AEFC, as well as in (N, O) *Ank* and *Enpp1* ^−/−^ AEFC in diluted concentrations. Localization of OPN, DMP1, and BSP was not different in cellular cementum of null models vs. controls (D–F, J–L, and P–R). Abbreviations: d = dentin; c = acellular cementum; p = periodontal ligament; b = bone. Scale bar = 100 µm.

**Figure 8:**
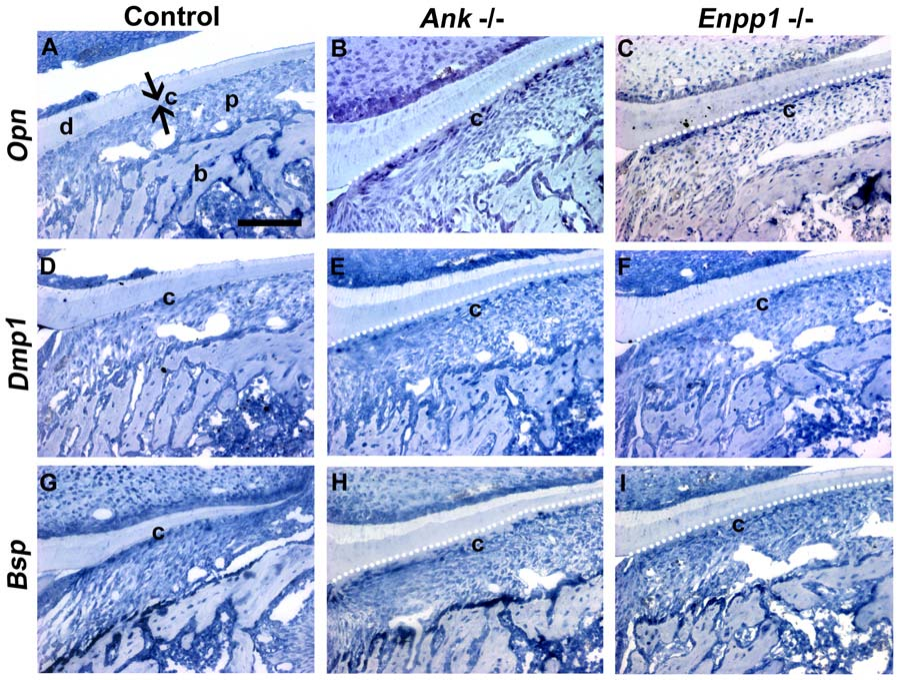
Reduced pyrophosphate alters gene expression in cervical cementoblasts. *Opn* mRNA is markedly increased in root-lining cementoblasts in both (B) *Ank* and (C) *Enpp1* ^−/−^, compared to (A) *Ank* and *Enpp1* ^+/+^ controls. Increased numbers of cells associated with the thick cervical cementum express *Dmp1* in (E) *Ank* and (F) *Enpp1* ^−/−^ molars, compared to (D) ^+/+^ controls. *Bsp* gene expression was not different in cementoblasts in (H) *Ank* and (I) *Enpp1* ^−/−^ vs. (G) ^+/+^ controls. Black arrowheads indicate regions of positively stained cells. All panels are samples from mice at 14 dpn. Abbreviations: d = dentin, c = (cervical) cementum; p = periodontal ligament; b = bone. Scale bar = 100 µm.

Cementum, bone, and dentin are also characterized by their extracellular matrix (ECM) protein composition, and these ECM proteins contribute to crystal growth and regulation, and affect mechanical properties of these tissues. Because of the dramatic changes in cementum apposition, we investigated the ECM profile in PP_i_ deficient mice. In the low PP_i_ environment of the *Ank* and *Enpp1* ^−/−^ mice, the thick cervical cementum was marked by increased OPN and dentin matrix protein 1 (DMP1), proteins of the SIBLING family (Figure 7A–F and G–L). OPN staining strongly labeled control acellular cementum, and was intensely expressed in the corresponding *Ank* and *Enpp1* ^−/−^ cervical cementum and associated cementoblast cells. DMP1, a marker for osteocytes, odontoblasts, and cementocytes, was present at low or undetectable levels in acellular cementum in controls, in contrast to intense localization in expanded *Ank* and *Enpp1* ^−/−^ cementum. OPN and DMP1 levels were not changed in *Ank* or *Enpp1* ^−/−^ apical cementum, as well as in other dentoalveolar locations. The source of the increased OPN and DMP1 protein was confirmed, as cementoblast gene expression for both *Opn* and *Dmp1* mRNA was increased in *Ank* and *Enpp* ^−/−^ mice (Figure 8A–C and D–F). OPN and DMP1 expression changes were not observed in other cell populations in the dentoalveolar complex in these mice. Another characteristic marker for cementum, BSP, was present in control and null cementum (Figure 7M–R), and where protein concentration was diluted in the larger cementum volume of the *Ank* and *Enpp1* ^−/−^ mice, mRNA levels in cementoblasts were unaltered (Figure 8G–I).

Thus, increased cementogenesis in *Ank* and *Enpp1* ^−/−^ teeth was linked to increased OPN and DMP1 specifically in cervical cementum. It is notable that OPN was increased in cementum as a result of reduced extracellular PP_i_. This change is opposite to the decreased OPN that has been documented in osteoblasts and articular locations in mice lacking ANK or NPP1 [14], [16].

### Cementoblasts express pyrophosphate regulators in a time and space restricted manner

Acellular cementum was shown to be exceptionally sensitive to regulation by PP_i_; with increased PP_i_ (as in *Alpl* ^−/−^ mice) AEFC was severely inhibited, and under reduced PP_i_ conditions (as in *Ank* and *Enpp1* ^−/−^ mice) cementum thickness increased significantly, a trend not reflected in other dental hard tissues. In order to understand the sensitivity of acellular cementum to PP_i_ metabolism, we mapped the expression of TNAP, ANK, and NPP1 during tooth root formation. We also assayed these factors in all of the null models to determine if there were compensatory or antagonistic expression changes that would contribute to phenotypes under PP_i_ dysregulation.

**Figure 9:**
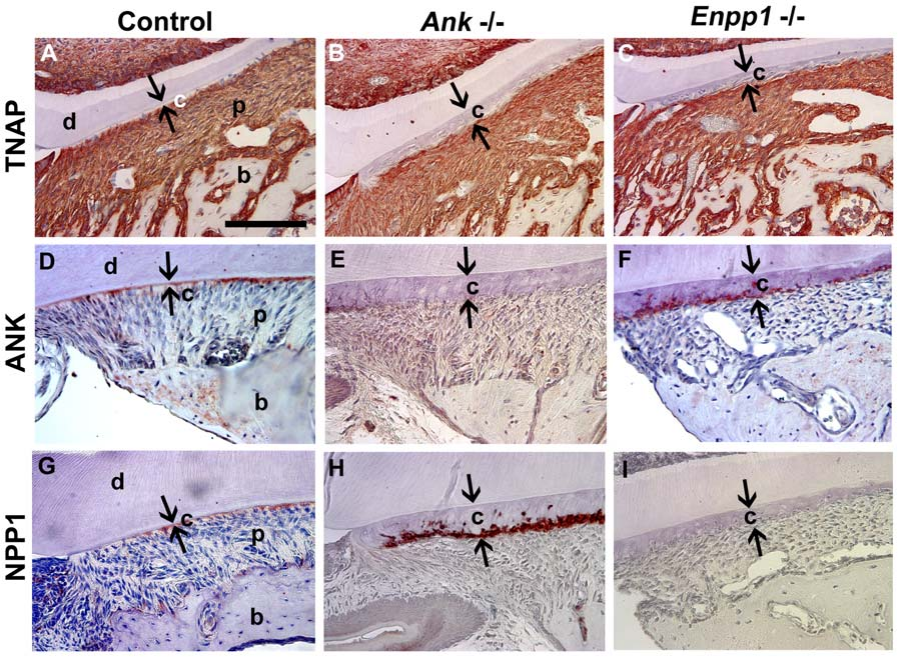
Cementoblasts express pyrophosphate regulators in a time and space restricted manner. TNAP was expressed strongly in all the periodontal tissues in (A) *Ank*; *Enpp1* ^+/+^ control as well as both (B, C) *Ank* and *Enpp1* ^−/−^ models. Loss of ANK or NPP1 did not alter cementoblast TNAP expression. ANK was localized selectively to cementoblasts in (D) control, and was increased when (F) *Enpp1* was ablated. Like ANK, NPP1 was found at selectively greater concentrations in cementoblasts in (G) controls, and was increased upon (H) *Ank* ^−/−^. Specificity of antibody staining was confirmed in null mice in (E) and (I). All panels are mandibular first molar teeth at 26 dpn. Abbreviations: d = dentin; c = acellular cementum; p = periodontal ligament; b = bone. Cervical cementum is indicated by opposing black arrows. Scale bar = 100 µm.

TNAP was widely expressed during molar root formation, most strongly in mineralizing osteoblasts, odontoblasts, and cementoblasts (Figure 9A). As previously reported, TNAP was also strongly localized to the PDL region [28], [29]. TNAP localization was not altered in developing *Ank* and *Enpp1* ^−/−^ molars (Figure 9B and C).

We previously reported wide expression of ANK gene and protein in the tooth and supporting tissues [26], paralleling previous findings that ANK is expressed in several tissues system-wide [12]. Using a refined immunohistochemistry technique, which allowed more sensitive identification of differential ANK protein localization, we discovered that after acellular cementum formed, ANK was labeled most intensely in cementoblasts lining the molar and incisor roots (Figure 9D). Developmental localization of NPP1 protein was similar to that of ANK, with most intense staining found in cementoblasts (Figure 9G). Both ANK and NPP1 stained weakly in other cells, including PDL cells, osteoblasts, and odontoblasts. Immunolocalization revealed compensatory up-regulation, where NPP1 was increased in *Ank* ^−/−^ and ANK was increased in *Enpp1* ^−/−^ (Figure 9F and H). Most interestingly, the observed increase was found only in cementoblasts, and not in other cell populations of the dentoalveolar region. These data suggested that ANK and NPP1 were differentially expressed by cementoblasts and employed to tightly regulate PP_i_ and developmental cementum apposition. However, it still remained unclear by what mechanism PP_i_ was controlling cementum apposition and ECM composition.

### Pyrophosphate controls mineralization and coupled gene expression in cementoblast cultures

**Figure 10:**
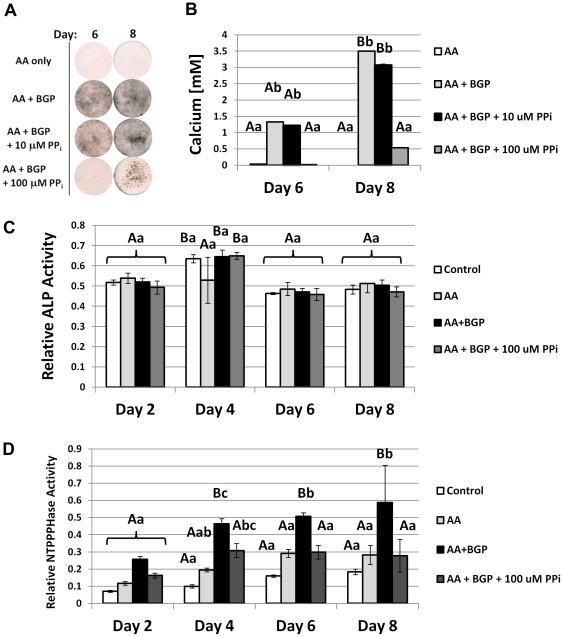
Pyrophosphate regulates cementoblast mineralization and nucleotide pyrophosphohydrolase (NTPPPH) activity, *in vitro*. (A) By von Kossa staining, OCCM.30 cells cultured with 5 mM BGP produced mineral nodules by days 6 and 8, while cells receiving only AA did not mineralize. The low dose of 10 µM PP_i_ did not affect mineral nodule precipitation, however, the higher dose of 100 µM was a potent inhibitor of mineral nodules. (B) Quantitative calcium assay performed on days 6 and 8 confirmed visual mineral nodule staining by von Kossa. (C) Relative ALP enzyme activity was not affected by inhibition of mineralization by 100 µM PP_i_. (D) NTPPPHase activity was increased under mineralizing conditions, but inclusion of 100 µM PP_i_ brought activity back to basal levels of non-mineralizing cultures. Graphs show mean +/− SD for n = 3 samples. Lowercase letters indicate treatment comparison at each time point, where different letters indicate a statistically significant intergroup difference. Uppercase letters indicate comparisons over time in the same treatment group, where different letters indicate a statistically significant intragroup difference. Values sharing the same uppercase or lowercase letter in were not significantly different. Means were compared by ANOVA (p<0.05) followed by the Tukey test for direct pair-wise comparisons.

PP_i_ regulators ANK and NPP1 were preferentially expressed by cementoblasts after initiation of cementogenesis, and their expression was modulated under conditions of low extracellular PP_i_ and increased apposition. Expression levels of cementum ECM proteins OPN and DMP1 were also responsive to PP_i_ deficiency, reflecting the altered homeostasis of P_i_/PP_i_ ratio or increased cementum apposition in *Ank* and *Enpp1* ^−/−^. These data together suggested that cementoblasts associated with AEFC regulate PP_i_ as a means to tightly control the process of apposition and related gene expression. *In vitro* experiments were performed to determine how these genes were regulated during mineral formation, and what potential role PP_i_ played in their regulation. Because of the technical obstacles in isolating and identifying primary cementoblasts, we opted to use an immortalized cementoblast cell line (OCCM.30) and modulate exogenously added PP_i_. OCCM.30 cells were cultured in control media or mineralization media where 5 mM β-glycerophosphate (BGP) was added. BGP served as an organic P_i_ source, mimicking similar sources *in vivo* and commonly used for *in vitro* mineralization experiments [30]–[33]. Cells receiving control media lacking BGP failed to mineralize during the course of the experiment. While cells cultured with BGP produced mineral nodules by day 6, with increased staining and calcium incorporation at day 8 (Figure 10A and B).

**Figure 11:**
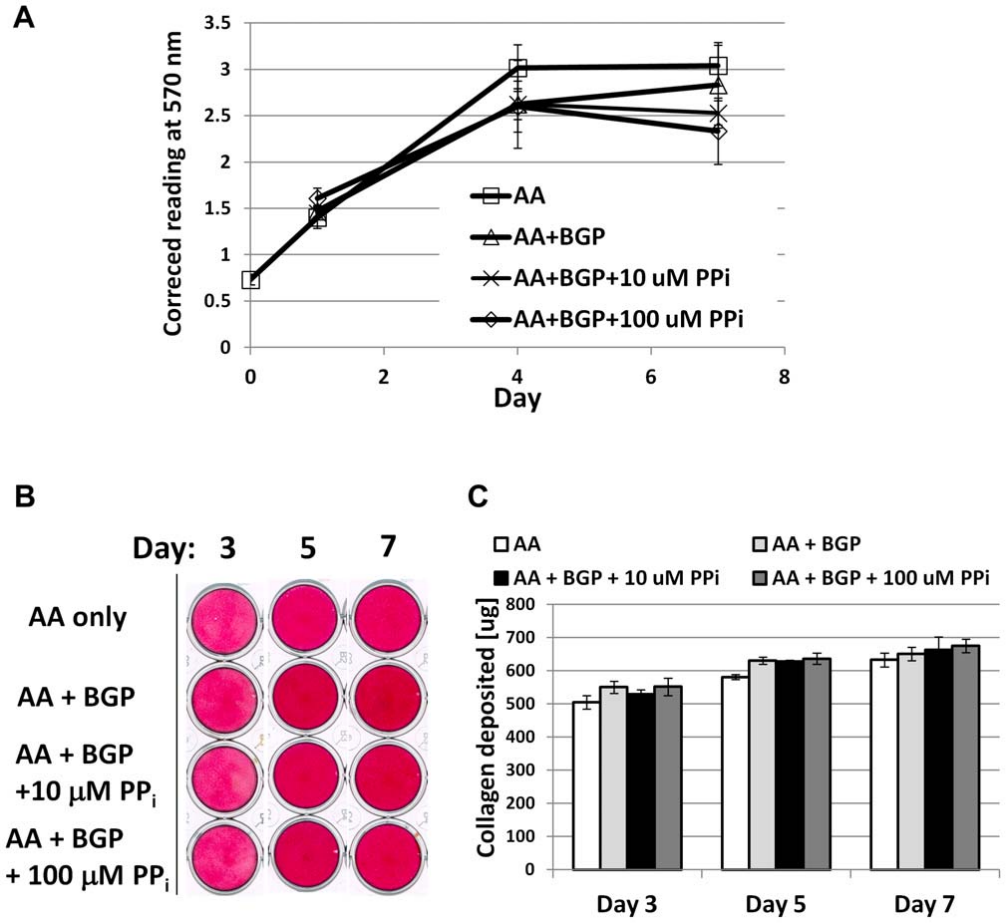
Pyrophosphate does not affect cementoblast proliferation or collagen synthesis, *in vitro*. (A) Cell proliferation was assayed by MTS assay where absorbance at 570 nm is proportional to the number of living cells in culture. No difference in OCCM.30 cementoblast cell number was found between non-mineralizing (AA) and mineralizing (AA + BGP) treatments at concurrent time points, including with doses of 10 or 100 µM PP_i_. (B) Picrosirius red dye was used to stain collagen deposited by cementoblasts at days 3, 5, and 7. (C) Quantification of the collagen-binding assay did not identify any treatment differences for collagen deposition at any of the time points. For both (A) and (C), graphs show mean +/− SD for n = 3 samples, and no intergroup significant differences (at the same time point) were identified by one-way ANOVA and post-hoc Tukey analysis, for α = 0.05.

Cells were introduced to exogenous PP_i_ to create culture conditions of low (10 µM) and high (100 µM) PP_i_. The lower dose of 10 µM PP_i_ did not affect mineralization, while the higher dose of 100 µM was confirmed as an inhibitor of mineral nodule formation under these conditions. While PP_i_ is an inhibitor of HAP crystal precipitation, it has also been reported to have cell signaling effects in osteoblasts [4], [14], [16]. Neither dose of PP_i_ affected OCCM.30 cell proliferation, viability, or collagen synthesis compared to controls (Figure 11), therefore these processes were not indirectly affecting mineralization. Cementoblast ALP enzyme activity was uniform across treatments and times, and added 100 µM PP_i_ did not appreciably affect ALP (Figure 10C), indicating the effect of PP_i_ on mineralization was not by inhibition of TNAP. An enzymatic assay for 5′-nucleotide phosphodiesterase I and nucleotide pyrophosphohydrolase (NTPPPH) activity demonstrated significantly increased NPP1 function with mineralization at days 4, 6, and 8, while 100 µM PP_i_ brought activity back to basal levels of non-mineralizing cultures (Figure 10D).

**Figure 12:**
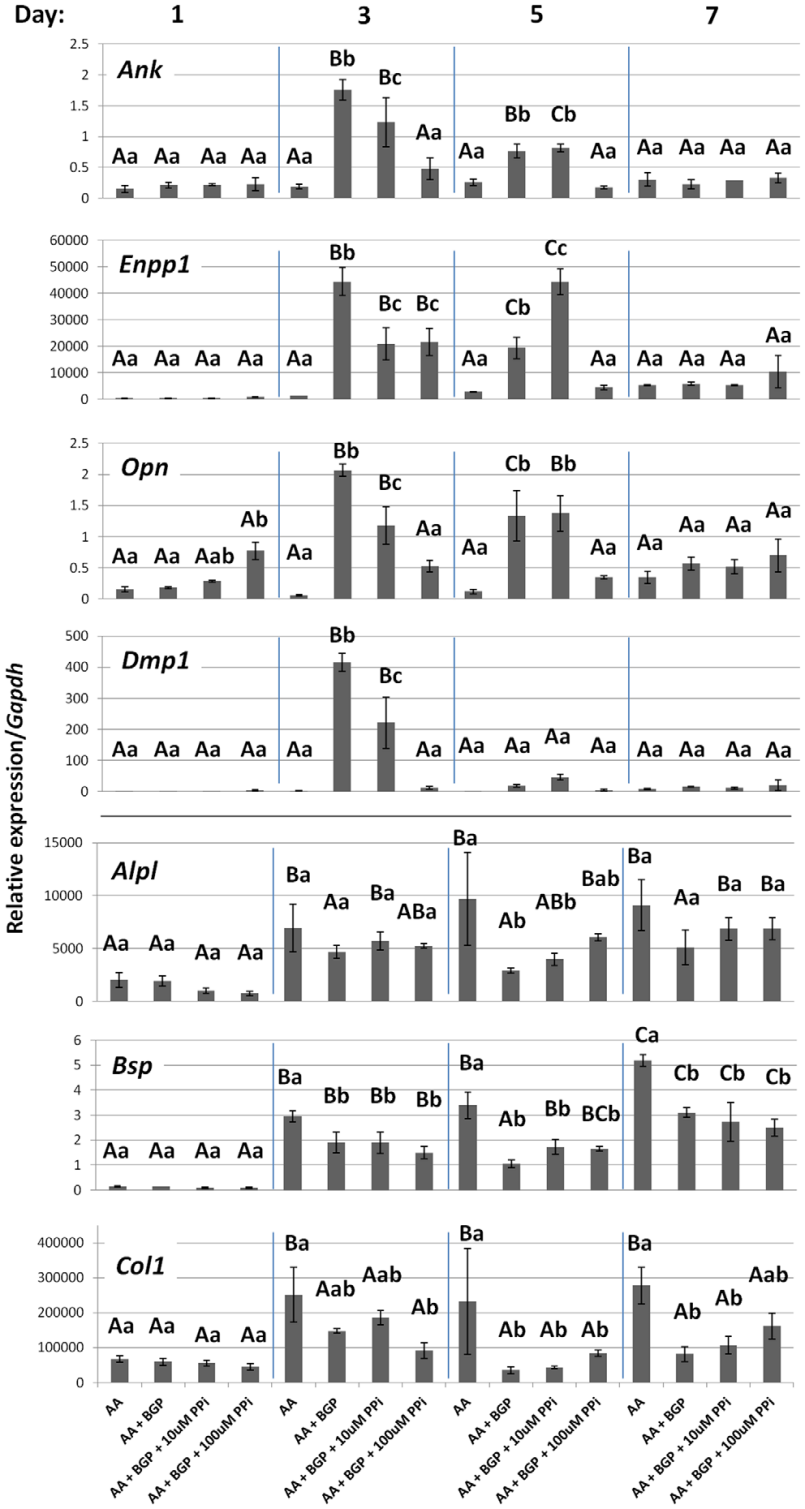
Pyrophosphate regulates cementoblast mineralization-coupled gene expression. Mineralizing cultures (AA + BGP) increased expression of *Ank*, *Enpp1*, *Opn*, and *Dmp1* at days 3 and 5, concurrent with mineralization. The higher dose of 100 µM PP_i_ significantly depressed expression of all four genes on day 3 compared to mineralizing cultures; *Ank*, *Enpp1*, and *Opn* suppression was maintained on day 5. The lower dose of 10 µM PP_i_ showed milder effects on all four genes at day 3. Differences were not maintained by day 7. Unlike expression of *Ank*, *Enpp1*, *Opn*, and *Dmp1*, where PP_i_ was able to block mineralization-associated induction, additional markers *Alpl*, *Bsp*, and *Col1* were not regulated in coordinated fashion by mineralization or inclusion of either dose of PP_i_. Graphs show mean +/− SD for n = 3 samples. Lowercase letters indicate treatment comparison at each time point, where different letters indicate a statistically significant intergroup difference. Uppercase letters indicate comparisons over time in the same treatment group, where different letters indicate a statistically significant intragroup difference. Values sharing the same uppercase or lowercase letter in were not significantly different. Means were compared by ANOVA (p<0.05) followed by the Tukey test for direct pair-wise comparisons.

PP_i_ associated and cementoblast marker genes were assayed by quantitative PCR. Under non-mineralizing conditions, *Ank*, *Enpp1*, *Opn*, and *Dmp1* did not change over the course of the experiment (Figure 12). However, all four genes increased significantly under mineralizing conditions at days 3 and 5, when mineral nodules were forming. At day 3, when increases were most dramatic, *Ank* increased almost 10-fold, *Enpp1* increased 30-fold, *Opn* increased more than 30-fold, and *Dmp1* increased 140-fold in mineralizing cultures compared to controls. These four genes also responded in parallel fashion to PP_i_. While inclusion of 10 µM PP_i_ had a mild effect on gene expression compared to ascorbic acid (AA) + BGP cultures (paralleling effects on mineralization), the higher dose of 100 µM PP_i_ significantly depressed *Ank*, *Enpp1*, *Opn*, and *Dmp1* expression on day 3 compared to mineralizing cells. Cells receiving the 100 µM dose also maintained significantly lower expression of *Ank*, *Enpp1*, and *Opn* on day 5. By day 7, expression levels of the four genes were low, and there were no differences between any of the treatment conditions. Other cementoblast marker genes assayed, including *Alpl*, *Bsp*, and *Col1*, did not show a coherent pattern in response to addition of PP_i_. Notably, the increase in *Enpp1* gene expression associated with mineralization corresponds to the increase in NTPPPHase activity recorded, and inclusion of PP_i_ decreased mineralization and correspondingly decreased *Enpp1* gene expression and NPP1 enzyme activity. In contrast, PP_i_ did not perturb cementoblast mineralization by affecting *Alpl* expression or ALP activity.

**Figure 13:**
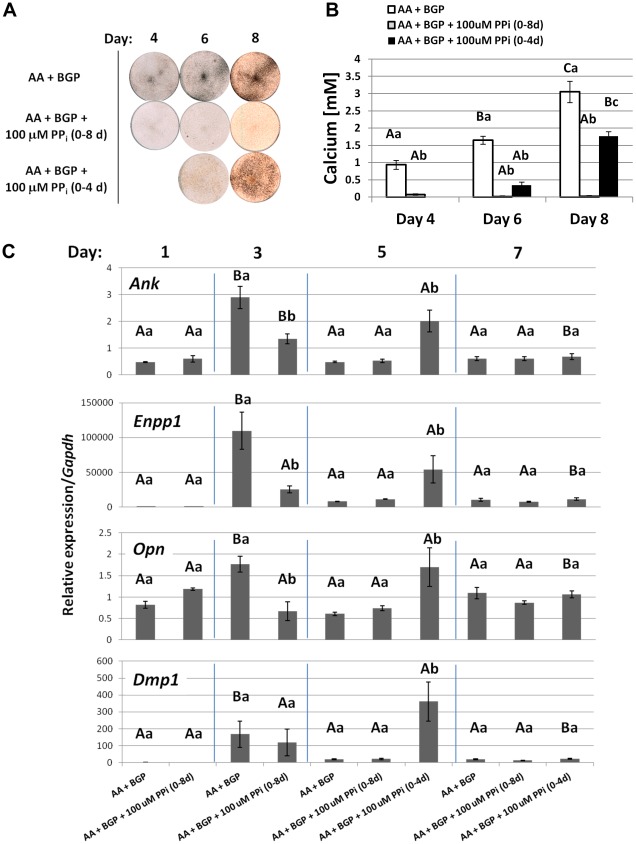
Timing of pyrophosphate removal determines cementoblast mineralization and coordinated gene expression *in vitro*. (A) By von Kossa staining, OCCM.30 cells cultured with 5 mM BGP produced mineral nodules by days 4, 6, and 8, while inclusion of 100 µM PP_i_ inhibited mineralization for the entire experiment. When PP_i_ was removed after 4 days, OCCM.30 cells began mineralizing the matrix by days 6 and 8. (B) Quantitative calcium assay performed on days 4, 6, and 8 confirmed visual mineral nodule staining by von Kossa. (C) Mineralizing cultures (AA + BGP) increased expression of *Ank*, *Enpp1*, *Opn*, and *Dmp1* at day 3, concurrent with mineralization. Inclusion of 100 µM PP_i_ significantly depressed expression of *Ank*, *Enpp1*, and *Opn* on day 3 compared to mineralizing cultures. Removal of PP_i_ on day 4 led to increased *Ank*, *Enpp1*, *Opn*, and *Dmp1* on day 5, coincident with mineralization. Graphs in (B) and (C) show mean +/− SD for n = 3 samples. Lowercase letters indicate treatment comparison at each time point, where different letters indicate a statistically significant intergroup difference. Uppercase letters indicate comparisons over time in the same treatment group, where different letters indicate a statistically significant intragroup difference. Values sharing the same uppercase or lowercase letter in were not significantly different. Means were compared by ANOVA (p<0.05) followed by the Tukey test for direct pair-wise comparisons.

These results showed that PP_i_ regulated cementoblast mineralization and associated gene expression, *in vitro*. In an additional experiment of similar design, the addition of 100 µM PP_i_ was discontinued in some wells midway through the experiment. Cells with 100 µM PP_i_ for the duration did not mineralize, while cultures relieved of PP_i_ inhibition at day 4 showed mineralization by day 6, increased *Ank*, *Enpp1*, *Opn*, and *Dmp1* by day 5, coincident with mineralization (Figure 13). This experiment demonstrated that even if PP_i_ inhibited initiation of mineralization for the first 4 days, its removal facilitated both mineralization and concomitant gene expression. These results support expression of *Ank*, *Enpp1*, *Opn*, and *Dmp1* as being functionally coupled to matrix mineralization, i.e. linked to changes in the mineralizing matrix. Importantly, these results parallel *in vivo* observations, where ANK, NPP1, OPN, and DMP1 were all increased by cementoblasts under conditions of reduced PP_i_, i.e. *Ank* or *Enpp1* ablation.

## Discussion

These studies aimed to define the regulatory role of PP_i_ in tooth root cementum development. We demonstrate here that PP_i_ serves as an essential regulator of tooth root acellular cementum development, and a key determinant defining the hard-soft interface between the cementum and PDL. Dysregulation of PP_i_ resulting from loss of any of the central PP_i_ controlling factors explored here had profound consequences on development of acellular extrinsic fiber cementum (AEFC), a tissue essential to tooth attachment and function. To wit, loss of TNAP caused severe underdevelopment or even absence of acellular cementum. Loss of either ANK or ENPP1 resulted in loss of control of cementum apposition, causing an exceptional hypercementosis. Because these three factors, TNAP, ANK, and NPP1, primarily adjust extracellular PP_i_, this strongly supports PP_i_ as the key mechanistic factor uniting the cementum phenotypes in all three of these mouse models, prompting us to propose that PP_i_ regulates acellular cementum in a molecular “rheostat” fashion, i.e. acellular cementum thickness relates inversely to PP_i_ production.

**Figure 14:**
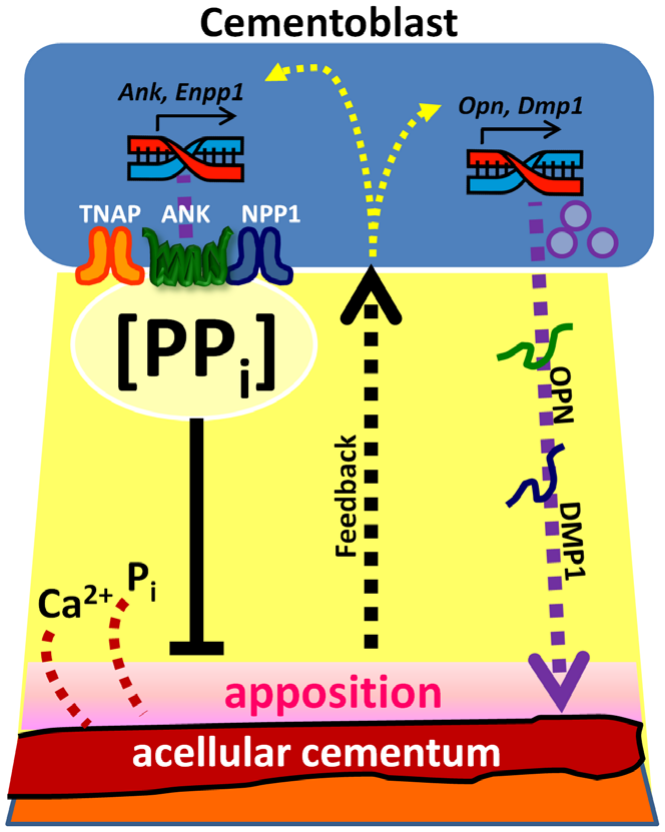
Model for the hypothesized role of in formation of acellular cementum. Cementum apposition depends on precipitation of calcium (Ca^2+^) and phosphate (P_i_) ions on the root surface, and pyrophosphate (PP_i_) acts as a potent inhibitor of hydroxyapatite crystal precipitation. Local pericellular PP_i_ concentration is controlled primarily by three cellular factors: Tissue nonspecific alkaline phosphatase (TNAP; hydrolyzes PP_i_ to P_i_), progressive ankylosis protein (ANK; regulates transport of PP_i_ from the intracellular to extracellular space), and ectonucleotide pyrophosphatase phosphodiesterase 1 (NPP1; produces PP_i_ from hydrolysis of nucleotide triphosphates). Cementoblasts express TNAP, ANK, and NPP1 in order to regulate local PP_i_ homeostasis and control the amount of cementum apposition. Cementoblasts are responsive to apposition by some type of outside-in feedback mechanism, and are capable of modulating expression of PP_i_ regulators as well as production of secreted matrix proteins such as OPN and DMP1, which may influence mineralization or properties of the matrix.

Based on these collective data, we propose a model whereby PP_i_ plays a central and novel role in acellular cementum formation (Figure 14). The periodontal region is extremely rich in ALP activity (reducing local PP_i_) and thus a permissive milieu for cementum formation on the root surface. In the course of normal development, cementoblasts modulate PP_i_ to curb apposition (by increasing PP_i_ via ANK and NPP1) to maintain AEFC as a thin tissue on the root surface. When one of these PP_i_ factors is removed from the equation, apposition cannot be fully regulated and cementoblasts attempt to compensate by increasing expression of its counterpart PP_i_ regulator. In addition to directly controlling cementum mineral apposition, these studies suggest PP_i_ influences ECM protein composition; in the face of rapid cementogenesis, cementoblasts increased expression of OPN and DMP1. The increase in OPN, a negative regulator of HAP crystal growth, may be an additional mechanism cementoblasts employ to limit extent of cementum apposition. *In vitro* experiments support this interpretation of the role of PP_i_ in controlling both mineral accumulation and cementoblast expression profile. What emerges is a portrait of acellular cementum as a mineralized tissue heavily governed by regulation of the physical-chemical process of mineral precipitation, and the cementoblast as a cell capable of directing PP_i_ metabolism to promote and restrain cementogenesis.

### On the role of pyrophosphate as a negative regulator of acellular cementum

In these studies, we confirm that loss of TNAP function in the *Alpl* ^−/−^ mouse causes aplasia or severe hypoplasia of the acellular cementum. This is in line with a previous report from this and another model of TNAP loss-of-function [23], [25], as well as reports from human hypophosphatasia (HPP) subjects [21], [34], who harbor a mutation in the human homologue, *Alpl* [7]. We extend previous analyses of the *Alpl* ^−/−^ tooth cementum phenotype with gene and protein assays. Cementoblasts express similar levels of *Bsp* mRNA, while protein distribution of both BSP and OPN appear disrupted. We interpret these results to mean that cementoblast phenotype is maintained in the face of loss of TNAP, but that disruption of AEFC synthesis prevents accretion of BSP and OPN proteins on the root surface. Loss of OPN protein under conditions where acellular cementum was inhibited has been reported previously [25], [35], and this observation makes sense because BSP and OPN are both mineral-binding members of the SIBLING family which play a role in the mineralization process [36], and in the close relationship of AEFC cementogenesis with the act of mineralization.

We show strong TNAP localization in the developing root region, and ALP activity has been reported to be strong in the periodontium, with highest activity adjacent to the mineralizing bone and developing cementum surfaces [29]. Moreover, the same study identified a significant correlation between measured TNAP activity and acellular cementum thickness. The critical influence of TNAP on cementum apposition is likely by clearance of mineralization inhibitor PP_i_, rather than by providing local P_i_ for HAP precipitation, for several reasons. First, circulating PP_i_ is in the micromolar range, while P_i_ is much higher in the millimolar range, so in the highly vascular periodontal region hydrolysis of PP_i_ is not likely to appreciably increase P_i_ available for mineralization, though local, compartmentalized ionic dynamics *in vivo* are difficult to predict. Secondly, mouse models of hypophosphatemia described to date tend to feature bone, dentin, and cellular cementum disorders, while acellular cementum is less affected [37], [38]. Thirdly, in studies employing a PP_i_ analog, 1-hydroxyethylidene-1, 1-bisphosphonate (HEBP), it was found that HEBP inhibited formation of acellular cementum entirely, while cellular cementum and bone matrices were produced, but remained unmineralized [35], [39], [40]. A parallel pattern emerged when mineralization inhibitor matrix gla protein (MGP) was ectopically expressed in bones and teeth; bone, dentin, and cellular cementum matrices were produced yet remained unmineralized, while AEFC was absent [41]; MGP and PP_i_ may have parallel functions as mineral regulators throughout the body. These studies indicate that cementogenesis depends heavily on creation of a physicochemical environment conducive for apposition, such as by PP_i_ clearance.

### Diminished pyrophosphate relieves the negative regulation on cementogenesis

Further evidence for PP_i_ as a central regulator of cementum thickness was garnered from studying models with deficient PP_i_, the *Ank* and *Enpp1* ^−/−^ mice. In these mice, a progressive thickening of AEFC was found during root development, which corroborated previous findings in mice harboring suspected loss-of-function mutations in these genes [42], [43]. By completion of root formation, these null models exhibited 12-fold or greater AEFC vs. controls, with a significantly increased rate of apposition over the developmental time period. Importantly, we have shown this expanded cervical cementum shares the same mineral and mechanical properties as WT controls [26], [44], [45]. This is strong evidence that PP_i_ is a key factor controlling acellular cementum formation, for several reasons. Firstly, ANK and NPP1 are membrane-bound proteins, which have been identified as primary regulators of extracellular PP_i_ concentrations around mineralizing cell types, as well as elsewhere in the body. However, they operate by different mechanisms, with ANK affecting PP_i_ transport and NPP1 acting as an ectoenzyme, producing PP_i_ through catalysis of trinucleosides. The common link in functions of both these proteins is extracellular PP_i_ production. That nearly identical AEFC phenotypes result from ablation of either of these genes is potent evidence for the indispensable role of PP_i_ in influencing acellular cementum formation. Though ANK and NPP1 share similarity in function by increasing extracellular PP_i_, loss of NPP1 causes a more severe skeletal hypermineralization phenotype in mice, a difference possibly related to inclusion of NPP1 in matrix vesicles, whereas ANK was found to be absent in matrix vesicles [16]. It is intriguing then that loss of ANK or NPP1 had nearly identical phenotypic results on acellular cementum, a tissue where there is no clear role of matrix vesicles in mineralization.

Secondly, ANK and NPP1 are expressed in the dentoalveolar region during tooth formation and cementogenesis. While both ANK and NPP1 are widely expressed throughout the body, both were found to be selectively more highly expressed in cementoblasts lining the tooth root. Also, a special importance for PP_i_ production in regulating cementum was indicated indirectly by findings that human PDL tissue expresses significantly higher basal levels of TNAP, ANK, and NPP1 than pulp [21], [46]. This hypothesis is supported by the finding that cementoblasts dramatically increased either ANK or NPP1 expression in response to loss of the other factor, likely an attempt to compensate for lack of extracellular PP_i_ output in these mice. The nature of the interaction between ANK and NPP1 in tooth formation is currently the subject of study in a series of double-deficient mice. That expression of these PP_i_ regulating factors is enriched in tooth root and they are inducible in each others' absence supports a central physiologic function for PP_i_ in normal control of cementogenesis. The possible involvement of other complementary and antagonistic factors in PP_i_ homeostasis in the root region is an intriguing question currently being studied. One candidate is CD73, a cell surface protein operating downstream of NPP1 which may regulate *Alpl* expression, and that has been linked to vascular calcification [47].

This essential role of PP_i_, however, seems to be limited to the cervical acellular cementum. ANK and NPP1 were not as consistently localized to regions of apical cementum, did not exhibit compensatory up-regulation in the apical portion of knock-out molars, and loss of ANK and NPP1 did not impact the phenotype of CIFC. In this respect, the cellular cementum showed a clear difference in developmental regulation from AEFC and more similarity to alveolar bone. Similarly, loss of TNAP and the resulting increased PP_i_ affected cellular cementum and bone in similar ways.

### On the influence of pyrophosphate metabolism on cementum extracellular matrix composition

Reduced PP_i_ not only resulted in more rapid AEFC apposition, but also led to altered cementoblast gene expression and matrix composition. OPN and DMP1, both mineral-regulating ECM proteins from the SIBLING family [36], were increased at the gene and protein level in *Ank* and *Enpp1* ^−/−^ cementoblasts. Expression of *Bsp*, a key cementoblast marker and SIBLING family member was unaltered in *Ank* and *Enpp1* ^−/−^ teeth. BSP immunostaining indicated a diffuse presence in the thick cementum, likely diluted in relatively greater volume of mineralized cementum. *Bsp* expression in cementoblasts *in vitro* was unaffected during mineralization or its inhibition by PP_i_.

OPN is a multifunctional ECM protein and a marker for cementum [48]–[50]. OPN has been shown *in vitro* to be an inhibitor of hydroxyapatite mineral crystal growth [51], [52], an observation supported by study of the *Opn* ^−/−^ (*Spp1* ^−/−^) mouse [53], as well as other models where increased OPN was found to disrupt skeletal mineralization [16], [54]. We suggest that increased expression of OPN by *Ank* and *Enpp* ^−/−^ cementoblasts represents an additional mechanism for control of apposition; like NPP1, an attempt at normalization of the cementogenesis process. While previous studies using osteoblasts have cited PP_i_ as a signal increasing *Opn* expression [4], [16], we found here that cementoblasts exposed to PP_i_ *in vitro* significantly reduced *Opn* expression during the mineralizing phase of the experiment. The cementoblast reaction to increase OPN in response to mineralization under low PP_i_ conditions, is opposite that of osteoblasts, which were found to reduce OPN in *Ank* and *Enpp1* ^−/−^ mice, contributing to the bone pathology. The divergent response underscores the unique mineral metabolism of cementum.

DMP1 is highly expressed in the osteocytes embedded in bone matrix, and is associated with maintenance of the lacunar-canalicular system of these cells [55]–[57]. DMP1 was increased in osteocytes in loaded bone, perhaps functioning in the mechanical response [58], [59]. In the context of increased cementum apposition in *Ank* and *Enpp1* ^−/−^ mice, we hypothesize that induction of *Dmp1* gene expression reflects rapid apposition and embedding of cervical root cementoblasts as cementocytes. The cementoblasts that direct AEFC normally remain as lining cells adjacent to this thin tissue. *In vitro*, mineralizing cementoblasts also increased *Dmp1* gene almost 140-fold, paralleling other studies where DMP1 was induced in periodontal ligament cells in mineralizing 3-dimensional gels [60].

The mechanism for altered *Opn* and *Dmp1* gene and protein expression is unknown, but under investigation. Based on *in vivo* and *in vitro* data, we propose an “outside-in” type of matrix-cell signaling mechanism whereby increased cementum apposition switches on expression of *Opn* and *Dmp1*, as well as *Ank* and *Enpp1*.

### Cementoblasts as pyrophosphate sensitive cells

Localization of PP_i_ regulators over the course of tooth development supported a role for PP_i_ in modulating cementogenesis. The developing periodontal region shows strong immunolocalization of TNAP and high ALP activity, in effect producing a highly pro-mineralization environment. These are favorable circumstances for apposition of the cementum layer on the root dentin surface. However, a question that arises is how cementum may remain a thin and slow growing mineralized layer in such an environment permissive for mineralization. ANK and NPP1 localized most strongly to the cementoblasts of the AEFC following cementum formation, suggesting initiation of cementogenesis occurs under the influence of TNAP activity, but that after cementum deposition (usually several µm in mice), cementoblasts increase ANK and NPP1 to restrict further cementum apposition. Thus, in a scenario where either ANK or NPP1 function is lost, cementum apposition is not adequately controlled and the other is up-regulated, along with increased OPN, in an attempt to regain homeostasis of cementum.

*In vitro* experiments employing a cementoblast cell line provided a mechanistic platform for probing these proposed roles of ANK, NPP1, and PP_i_ in cementoblast mineralization and gene expression. The response for cementoblasts to increase ANK, NPP1, and OPN in light of rapid apposition was supported by *in vitro* experiments employing a cementoblast cell line. Results from the studies described here demonstrated that gene expression of PP_i_ regulators *Ank* and *Enpp1* and ECM proteins *Opn* and *Dmp1* are functionally coupled to the mineralization process, increasing under mineralizing conditions and coincident with mineral nodule formation. Under conditions of higher PP_i_, mineral apposition was hindered, and expression of *Ank*, *Enpp1* (and *Opn* and *Dmp1*) was blocked in dose-response fashion. These *in vitro* data together with *Ank* and *Enpp1* ^−/−^ mouse phenotypes, as well as ANK and NPP1 tissue localization, support the hypothesis that PP_i_ modulation is employed by cementoblasts to guide the relative amount of cementum formed.

The question can be raised as to whether or not these experiments were carried out within a relevant physiological range of PP_i_. Circulating PP_i_ in normal individuals has been measured in the low µM range. We carried out experiments with 10 and 100 µM added PP_i_, and justified these doses based on the following considerations. Firstly, our primary interest was to determine if PP_i_ at a dose that could inhibit mineralization would also influence cementoblast gene expression. Using these cells under these culture conditions, the lower dose of 10 µM PP_i_ was insufficient in blocking mineralization in these cells. Mineralization is affected by multiple factors including cellular activities, cell culture media, Ca^2+^ and P_i_ availability, as well as known and unknown factors present in fetal bovine serum (FBS) supplemented to the media; therefore, it is difficult to directly compare doses across studies where one or more of these variables may differ, especially cell type and time points examined [4], [16]. Based on these criteria, we employed the higher dose of 100 µM PP_i_ to create high PP_i_ conditions for cells. Secondly, while circulating PP_i_ levels are reported, it remains unknown how local, pericellular concentrations of PP_i_ may vary. Biomineralization is well known to be a process dependent on compartmentalization, i.e. creation of localized, protected regions conducive to mineral precipitation. Therefore, we reasoned that cementoblasts, cells that show high *in vivo* and *in vitro* expression of PP_i_ regulators ANK and NPP1, could potentially create localized, high PP_i_ conditions to mediate biomineralization-related activities. This hypothesis supported using a higher exogenous dose of PP_i_ in order to effectively regulate OCCM.30-mediated mineralization *in vitro*.

### Insights into acellular and cellular cementum development and regeneration

In mammals, the fibrous connection of the tooth to the bony socket is classified as a gomphosis, or fibrous joint, and is unique in the body in that the periodontal ligament joins bone on one side to a non-bone substance on the other side, in this case the tooth root cementum [61]. The gomphosis attachment is unique to mammals and crocodilian reptiles, having developed from more ancient forms of tooth attachment such as direct ankylosis to bone [62]–[65]. This interposed ligament was made possible likely by a combination of events such as alterations in the HERS during root development, changes in the supporting bone (“bone of attachment”), and the rise of a unique tissue, the cementum, as well as diminution of mineralization in the region between bones and teeth [66]. The development of a mineral-free PDL region between mineralized bones and teeth requires localized expression of factors to establish a mineralization boundary at the hard-soft tissue interface and continue to maintain PDL space throughout the life of the tooth. In these studies, we demonstrate that maintenance of that hard-soft interface at the tooth root surface depends on finely tuned PP_i_ homeostasis. Intriguingly, unlike the ectopic calcification found in the joints, the PDL remained nonmineralized in the face of cementum expansion in *Ank* and *Enpp1* ^−/−^ mice, indicating the presence of additional factors that inhibit mineralization across the PDL space. These likely include multiple, redundant negative regulators of mineral growth, as well as factors that indirectly prevent mineralization by influencing cell differentiation [67].

These studies lend support to the idea that there are key differences influencing acellular versus cellular cementum development. Namely, acellular cementum is dependent on precise modulation of local PP_i_, whereas cellular cementum is much less sensitive to fluctuations in local PP_i_. The morphology, speed of formation, and ECM protein composition of acellular cementum were altered dramatically by disruption of local PP_i_ homeostasis. Conversely, cellular cementum remained unaffected in all of these aspects. The primary cementoblasts of the AEFC adapted their expression of ANK, NPP1, and OPN in attempts to compensate for loss of control over cementum apposition, a response notably absent in cementoblasts of cellular cementum, as well as other cells of the dentoalveolar complex. However, when the regulatory influence of PP_i_ was lessened, as in *Ank* and *Enpp1* ^−/−^ mice, the cementum of the cervical root grew rapidly, engulfed cells to become cementocytes, and switched on DMP1, approximating several aspects of apical cellular cementum. Thus, we propose that strict regulation by PP_i_ is one of the major differences between acellular vs. cellular cementum types.

This finding provides insight into the origins of the two main types of cementum, and also informs clinical regenerative therapies. The profound influence of PP_i_ metabolism on acellular cementum development immediately suggests the concept of PP_i_ modulation for cementum regeneration. It is especially attractive to consider such a novel approach when growth and differentiation factors used to date have been limited in terms of true cementum regeneration, PDL integration, and/or predictability [68]–[70]. In a preliminary proof-of-principle study, we employed the *Ank* ^−/−^ mouse, featuring deficient extracellular PP_i_ and increased cementogenesis, to analyze tissue repair and regeneration in a periodontal fenestration model [45]. Importantly, we found that *Ank* ^−/−^ mice featured significantly greater new cementum vs. controls, more organized mineral deposition on the root surface in the defect areas, and recapitulated expression patterns mapped during cementum development, including strong OPN and DMP1 in the cementum matrix, and elevated NPP1 in associated cementoblasts. Thus, in this pilot study in mice we found that reduced local levels of PP_i_ promoted increased cementum regeneration. There has been concern voiced about regenerated cementum being the cellular type in a majority of studies [as summarized in [69]], and thus not optimal for PDL attachment. Our findings support that the cellular or acellular nature may be a reflection of the speed of formation, and that both can support sufficient extrinsic PDL fiber insertion.

## Materials and Methods

### Ethics Statement

This study was performed in accordance with the recommendations in the Guide for the Care and Use of Laboratory Animals of the National Institutes of Health and AVMA Guidelines on Euthanasia. The protocol for all animal studies was approved by the Institutional Animal Care and Use Committee (IACUC), University of Washington, Seattle, WA (Protocols 4010-01 and 4010-03).

### Mouse strains

Preparation and genotyping of mouse models was previously described for *Ank* ^−/−^ [26], [71], *Alpl* ^−/−^ (previously known as *Akp2* ^−/−^), and *Enpp1* ^−/−^ [10], [14], [16], [54]. *Ank* and *Alpl* mice were maintained on a mixed background of 129S1/SvImJ and C57BL/6 strains, and *Enpp1* mice were maintained on a mixed background of C57BL/6×129/SvTerJ strains. Mice were housed in a specific pathogen free facility in 12 hr light-dark cycles with access to water *ad libitum*. *Ank* and *Enpp1* ^−/−^ mice were fed a standard rodent diet, while *Alpl* litters were provided a vitamin B6 enforced diet to reduce seizures and prolong lifespan (TestDiet, Richmond, IN). Heterozygote breeding pairs were employed to prepare homozygote ^−/−^ mice and age-matched ^+/+^ controls at specific ages during tooth development. Heterozygotes were examined to determine any morphological tooth phenotype. Mice were sacrificed by cervical dislocation and mandible tissues harvested. At least three control (^+/+^) and null (^−/−^) mandibles were examined for each age of interest.

### Histology

Mouse mandibles were harvested and prepared for histology as previously described [26]. Briefly, mandibles were sagittally hemisected and fixed in Bouin's solution at 4°C overnight. Hemi-mandibles were demineralized (for tissues post 8 dpn) in acetic acid/formalin/sodium chloride (AFS) solution at 4°C. Tissues were paraffin embedded after standard histological processing. Five µm buccal-lingual (coronal) serial sections of the first mandibular molar or longitudinal (sagittal) sections of hemi-mandibles were prepared by rotary microtome and mounted on charged glass slides. Slides were deparaffinized in xylene for histological analyses, including hematoxylin and eosin (H&E) staining used for morphological characterization.

### Growth measurement and histomorphometry

Cervical cementum on the lingual aspect of the mesial root of the mandibular first molar was measured at a fixed distance of 300 µm from the cementum-enamel junction (CEJ) in *Ank* and *Enpp1* ^+/+^ and ^−/−^ sections from 14–60 dpn, using two central sections from the set of serial sections. Static histomorphometry was used to measure cervical cementum, PDL, and alveolar ridge bone width on the lingual aspect of mesial roots of mandibular first molars at the age 26 dpn. Calibrated measurements were made using SPOT software (Diagnostic Instruments, Sterling Heights, MI). ANOVA followed by the Tukey test for direct comparisons was employed for statistical testing of histomorphometric measurements (PASW (SPSS) Statistics software, version 19).

### Picrosirius red stain for collagen in histological sections

Tissues processed for histology were stained with a picrosirius red staining kit according to manufacturer directions (Polysciences, Inc., Warrington, PA). Deparaffinized slides were immersed in 0.2% phosphomolybdic acid hydrate, rinsed in water, incubated in direct red 80 for 60 min, then 0.01 N HCl solution for an additional 2 min. Samples were rinsed in 75% ethanol for 45 sec, then dehydrated in xylene, cleared, and mounted with coverslips. Digital images were captured with an OptiPhot-2 microscope (Nikon Instruments, Inc., Melville, NY) fitted with a light polarizer, using an EOS 5D Mark II digital camera (Canon U.S.A., Inc., Lake Success, NY).

### Immunohistochemistry

Immunohistochemistry (IHC) was performed on histological sections as previously described [26]. Primary antibodies were used with biotinylated secondary antibodies (Vectastain Elite ABC, Vector Labs, Burlingame, CA) and color reactions were developed to a red product using a 3-amino-9-ethylcarbazole (AEC) substrate kit (Vector Labs). Positive controls included normal mouse tissues and negative controls were performed in the absence of primary antibody. Primary antibodies included: monoclonal rat anti-human ALPL/TNAP (R&D Systems, Minneapolis, MN); rabbit anti-mouse progressive ankylosis protein (ANK3) [12]; rabbit anti-mouse bone sialoprotein (BSP), (a gift from Dr. Renny Franceschi, University of Michigan); rabbit anti-rat dentin matrix protein-1 (DMP1) raised against an N-terminal (90–111) portion of DMP1 (Takara, Shiga, Japan); polyclonal goat anti-human NPP1 (Abcam, Cambridge, MA); and LF-175 rabbit anti-mouse osteopontin (OPN) (Dr. Larry Fisher, NIDCR) [72]. ALPL and ANK3 staining was performed with an additional unmasking step wherein slides were incubated overnight in 8.0 M guanidine HCl (pH 8.0) solution. IHC for each target protein was performed in sections from at least three (n = 3) animals for each age, with representative staining chosen for photographs shown in Results.

### *In situ* hybridization

*In situ* hybridization was performed using a non-radioactive *in situ* hybridization (ISH) protocol employing a digoxigenin (DIG)-labeled cRNA probe for genes of interest, as described previously [26]. Linearized probes were cleaned by phenol-chloroform precipitation. Riboprobe synthesis was performed using a digoxigenin–UTP-labeled kit (Roche Applied Science, Indianapolis, IN). Probes were fractionated at 60°C and riboprobe concentrations were checked by dot blot on Hybond N^+^ nylon membrane (GE Healthcare, Piscataway, NJ). Messenger RNAs were labeled by incubation of deparaffinized sections with NBT/BCIP (Nitro blue tetrazolium chloride/5-Bromo-4-chloro-3-indolyl phosphate, toluidine salt). Probes used for ISH included: mouse *Dmp1* plasmid (provided by Dr. Ann George, Northwestern University) [73]; mouse *Opn* and *Bsp* probes (provided by Dr. Marian Young, NIH/NIDCR) [74]. Negative controls included sense probes.

### Electron microscopy

Scanning electron microscopy (SEM) analyses were performed on hemi-mandibles from 20 dpn control and *Alpl* ^−/−^ mice as previously described [26]. Briefly, mandibles were sequentially dehydrated in aqueous ethanol solutions and mounted in room-temperature-cure epoxy (Allied High Tech Inc, Rancho Dominguez, CA). Specimens were cut using a precision wafering saw (Buehler Ltd, Lake Bluff, IL) to expose the mesial surface of the first molar. The cut surface was then ground further distally to expose the interior of the first molar using 600 then 1500 grit SiC papers, followed by smoothening via ultramicrotoming with a 45° angle diamond knife (Diatome, Inc., Hatfield, PA) fitted onto a MT 6000-XL ultra-microtome (Bal-Tec RMC, Inc., Tucson, AZ). Specimens were mounted on SEM stubs, sputter coated with 5 nm of Pt for electron conductivity (SPI Supplies Inc, West Chester, PA), and imaged by an JSM7000F (JEOL-USA, Inc., Peabody, MA) SEM operating at 15 kV in backscattering mode.

### Cell culture and *in vitro* assays

Isolation and characterization of OCCM.30 murine cementoblasts has been previously described [75], [76]. Cells were grown in Dulbecco's Modified Eagle Medium (DMEM) with 10% v/v fetal bovine serum (FBS), 2 mM L-glutamine, 100 U/ml penicillin, and 100 µg/ml streptomycin (all reagents from Invitrogen, Carlsbad, CA). For gene expression and mineralization experiments, OCCM.30 cells were plated in standard media as described above, with media changed after 24 hrs to DMEM with 1% FBS with 50 µg/ml ascorbic acid (AA). Media were changed every 48 hrs for the remainder of the experiment. Inclusion of organic phosphate source β-glycerophosphate (βGP; 5 mM) was used to create mineralizing conditions. Inorganic PP_i_ (10 or 100 µM) was added to assay effects on cell function. Both βGP and PP_i_ were purchased from Sigma-Aldrich (St. Louis, MO). Cell culture experiments were performed at least three times in triplicate with representative results presented.

Cell proliferation was measured using a non-radioactive, MTS-based assay, following manufacturer's directions (CellTiter 96® AQ_ueous_ proliferation assay, Promega, Madison, WI). Absorbance was measured at 570 nm, with reference reading at 750 nm. Absorbance is proportional to the number of living cells in culture.

Production of collagen by cells *in vitro* was quantified by picrosirius red staining, using methods modified from previous reports [77], [78]. Briefly, cells were rinsed with PBS and fixed in Bouin's solution for 1 hr at room temperature. The fixative was removed and the plate rinsed several times in water to remove excess Bouin's solution. The collagenous matrix in plates was stained by incubation with picrosirius red dye (Direct Red 80, Polysciences, Inc., Warrington, PA) while gently shaking. Unbound dye was removed by rinsing several times with 0.01 N HCl. Bound dye was removed by incubation and shaking with 0.1 N NaOH for at least 1 hr. Picrosirius red was quantified by reading the absorbance at 550 nm. Quantity of collagen was calibrated against a standard curve created by plating and eluting known concentrations of rat tail collagen.

Von Kossa staining for mineral nodule formation was performed using standard procedures [79]. Silver stain was visualized as black, and stain intensity indicated the amount of calcium phosphate precipitation in the cell matrix (silver ions react with phosphate). Cell mineralization *in vitro* was quantitatively assayed by measuring calcium deposits, using a method modified from a previous report [77]. To cell culture wells, 500 µl 0.5 N HCl was added and plates were agitated for 60 min to dissolve calcium-phosphate precipitations. Eluted calcium was measured using a calcium assay (Genzyme Diagnostics, Farmingham, MA). One µl of sample was added to 99 µl Arsenazo reagent and absorbance was read at 650 nm. Standard curves were prepared using a calcium stock solution.

A modified assay for measuring *in vitro* alkaline phosphatase activity (ALP) was used [80]. Briefly, cell cultures were rinsed with PBS and incubated with 200 µl p-Nitrophenyl phosphate (PNPP, Sigma) in the dark at ambient room temperature for 30 min. After incubation, 10 µl supernatant for each condition was transferred to a 96 well plate containing 90 µl of 3 N NaOH (stop solution) per well. Absorbance was recorded at a wavelength of 405 nm.

An enzymatic assay was employed to measure the activity of pyrophosphate-generating ectoenzymes (nucleoside triphosphate pyrophosphohydrolase, NTPPPHase activity), based on a previously described procedure [81]. Cells were rinsed with PBS and incubated for 2 hrs with 2.0 ml of 1 mM thymidine 5′ monophosphate p-nitrophenyl ester sodium (TMPNP) solution at 37°C and without CO_2_. After incubation, 20 µl supernatant for each condition was transferred to a 96 well plate containing 80 µl of 0.1 N NaOH (stop solution) per well. Absorbance was recorded at a wavelength of 410 nm.

### RNA isolation and real-time quantitative RT-PCR

Isolation of RNA, synthesis of cDNA, and performance of real-time quantitative PCR was undertaken as previously described [26]. Total RNA from cells was isolated using the RNeasy Micro kit (Qiagen, Valencia, CA) and cDNA was synthesized from 1.0 µg RNA (Transcriptor kit, Roche Applied Science). PCR reactions were performed with DNA Master SYBR Green I kit (Roche Applied Science) on the Roche Lightcycler 480 system (Roche Diagnostics GmbH, Mannheim, Germany) using intron-spanning primers (http://www.gene-expression-analysis.com/). Glyceraldehyde-3-phosphate dehydrogenase (*Gapdh*) was employed as a housekeeping/reference gene for target gene normalization and relative quantification with amplification efficiency correction. Primer sequences used are listed in Table 1. PCR product identification was performed by post-amplification melting curve analysis. To detect intra- and intergroup gene expression differences, we employed a one-way ANOVA with post-hoc Tukey test, using PASW (SPSS) Statistics 19 software.

**Table 1:** Real time quantitative PCR primer sequences.

Gene Symbol	Gene Name	Forward (5′–3′)	Reverse 5′–3′	Gene ID
*Alpl*	Tissue nonspecific alkaline phosphatase	GGGGACATGCAGTATGAGTT	GGCCTGGTAGTTGTTGTGAG	11647
*Ank*	Progressive ankylosis protein	GAATCAGTCGGCCCAT	GTTCGCCAGTTTATTGCT	11732
*Bsp*	Bone sialoprotein	GAGACGGCGATAGTTCC	AGTGCCGCTAACTCAA	15891
*Col1*	Collagen type 1 alpha 1	CACCCCAGCCGCAAAGAGT	CGGGCAGAAAGCACAGCACT	12842
*Dmp1*	Dentin matrix protein 1	GCGCGGATAAGGATGA	GTCCCCGTGGCTACTC	13406
*Enpp1*	Ectonucleotide pyrophosphatase phosphodisetrase 1	CGCCACCGAGACTAAA	TCATAGCGTCCGTCAT	18605
*Gapdh*	Glyceraldehyde-3 phosphate dehydrogenase	ACCACAGTCCATGCCATCAC	TCCACCACCCTGTTGCTGTA	14433
*Opn*	Osteopontin	TTTACAGCCTGCACCC	CTAGCAGTGACGGTCT	20750
